# Circulating microRNAs in breast cancer and healthy subjects

**DOI:** 10.1186/1756-0500-2-89

**Published:** 2009-05-19

**Authors:** Weizhu Zhu, Wenyi Qin, Ulus Atasoy, Edward R Sauter

**Affiliations:** 1 Department of Surgery, University of North Dakota, Grand Forks, ND, USA; 2 Department of Surgery, University of Missouri, Columbia, MO, USA

## Abstract

**Background:**

It has been demonstrated that extracellular mRNA can be detected in the circulation. Our hypothesis was that circulating miRNAs are also present and differentially expressed in the serum of breast cancer patients compared to controls.

**Findings:**

We measured miRNA in the serum of samples with and without the addition of miRNA prior to analysis. To test our RNA extraction efficiency, we spiked-in serial dilutions of single-strand C elegens miR-39 (cel-miR-39) and human miR-145 (has-miR-145) into goat serum and a 10 year old human serum specimen. We next analyzed miR-16, -145, and -155 in archived serum specimens from 21 participants, 13 of whom did and 8 of whom did not have breast cancer. We were able to detect the miRNAs from all the serum samples to which the miRNAs had been added. We were also able to detect endogenous miR-16, -145, and -155 in all serum samples. While the expression of all three miRNAs was similar in samples from healthy women compared to those with breast cancer, women with progesterone receptor (PR, p = 0.016) positive tumors had higher miR-155 expression than tumors that were negative for these receptors.

**Conclusion:**

1) RNA species can be detected in archived serum; 2) miR-155 may be differentially expressed in the serum of women with hormone sensitive compared to women with hormone insensitive breast cancer. Screening serum for miRNAs that predict the presence of breast cancer is feasible, and may be useful for breast cancer detection.

## Background and research hypothesis

There is increasing evidence supporting microRNA (miRNA) analysis for breast cancer diagnosis, prognosis and therapy [1-3]. miRNAs are ~22 nucleotide long RNA molecules encoded in the genome that can control gene expression. Their expression levels vary greatly among species and tissues [4]. Over 700 miRNAs have been identified from human tissue. Some of these short, endogenous non-protein coding miRNAs are linked to human malignancy and are differentially expressed in human cancer vs. matched normal tissue [5]. For example, a recent publication identified 29 miRNAs that were aberrantly expressed in primary breast cancer tissue. Of the 29, miR-10b, miR-125b, and miR-145 were down-regulated, while miR-21 and miR-155 were up-regulated [6]. Depending on the genes that the miRNAs act on, they may serve to stimulate or suppress tumor formation and growth. Another team reported 15 miRNAs that are expressed in a variety of normal human tissues. Among them, miR-16 is expressed at a higher level than the remaining 14 miRNAs [7]. These differences provide the molecular basis and rationale for the identification of miRNAs in breast cancer detection.

Cancer-related RNAs are present and detectable in serum [8-11], and it was recently reported that the levels of cyclin D1 and thymidylate synthase levels of free mRNA in plasma correlated with nonresponse to breast cancer therapy [12]. As RNase is present in the blood of healthy and cancer containing individuals [13], one would expect all circulating RNA (including miRNAs) in the blood to be rapidly destroyed. However, accumulated data suggest that circulating RNA may not be as fragile as was previously assumed. Tsui *et al*. reported that RNA in serum is more RNase resistant and stable than previously thought [14]. For example, tyrosinase mRNA has been found in the serum of melanoma patients but not in healthy individuals [9], and epithelial mRNA in the plasma of breast cancer patients was associated with poor prognosis [15]. Both observations demonstrate that cancer-related mRNA exists in the circulation and is detectable. Finally, a recent pilot study demonstrated that miRNAs can be detected in the serum of ovarian cancer patients [16]. Our hypothesis was that circulating miRNAs are also present and detectable in the serum of breast cancer patients.

## Methods

### Blood Collection and serum preparation

After informed consent was obtained from 21 participants (13 with breast cancer and 8 normal controls, Table 1), 6 ml of venous blood was collected from the antecubital fossa and placed in a serum separator tube. The blood was centrifuged at 1600 rpm for 5 min and the serum aliquoted into 1.7 ml Eppendorf tubes, followed by a 15 min high speed centrifugation at 12,000 rpm to completely remove cell debris, leaving only circulating RNA. To test our RNA extraction efficiency while minimizing possible interference from endogenous RNA, we used a 10 year old human serum sample and goat serum, to which cel-miR-39 and has-miR-145 were concurrently spiked in.

**Table 1:** Demographics of Participant Samples

	**Yes**	**No**	**Unknown**
**Breast Cancer**	13	8	

**Disease Stage**			

1	4		

2	5		

3	4		

**Estrogen receptor positive**	10	2	1

**Progesterone receptor positive**	9	3	1

**HER2/*neu *positive**	1	11	1

**Race**			

Caucasian	19		

African American	2		

### RNA Extraction

We first evaluated our RNA extraction efficiency. Either 10^5^, 10^3^, 10, 10^-1^, or 0 pg of single stranded cel-miR-39 and has-miR-145 synthesized by Invitrogen (Carlsbad, CA 92008) were spiked into 400 μL of both goat serum (Abcam, Cambridge, MA) and 10 year old archived human serum collected from a single subject. We isolated total RNA which included miRNA in 400 μL of serum from 21 participants using the MagMAX™Viral RNA Isolation kit following the manufacturer's instructions (Ambion, Austin, TX). The RNA was stored at -80°C until use. Both MagMAX™Viral RNA Isolation kit and mirVana paris kit ™ have been recommended to isolate small RNAs in serum by the supplier. We chose the MagMAX kit because it provided better miRNA recovery from the serum samples. We were able to increase our miRNA recovery by 1) extending the incubation and binding time from four to 10 minutes, and 2) increasing the ratio of isopropanol to wash buffer # 1 in the kit from 1:2 to 1:1.

### Reverse Transcription (RT) and Quantitative PCR (qPCR)

RT and qPCR kits made specifically for accurate miRNA analysis (Applied Biosystems) were used to evaluate expression of the following miRNAs from serum samples: cel-miR-39, has-miR-16, miR-145 and miR-155. 18s rRNA was used to normalize the RNA input. The RT reaction for 18s rRNA was performed with RETROscrit^® ^Kit (Ambion, catalog # AM1710). An equal volume of the eluted RNA (5 μL/reaction = 1/10 of the eluted volume) was reverse-transcribed with specific looped RT primers for each miRNA evaluated. The 15 μL RT reactions were performed using a TaqMan^® ^microRNA Reverse Transcription Kit (Applied Biosystems catalog # 4366596) and incubated for 30 min at 16°C, 30 min at 42°C, 5 min at 85°C, and then maintained at 4°C. For real-time PCR, 5 μL RT products were used as templates in 20 μL reactions containing primers and probes for each miRNA and 18s rRNA according to manufacturer instructions. All reactions were run on the MyiQ™ Real-Time PCR Detection System (Bio-Rad, Hercules, CA) using the following conditions: 95°C for 10 min, followed by 40 cycles at 95°C for 15 s, and 60°C for 1 min. The relative miRNA quantity in serum from participants with vs. without breast cancer was determined using the comparative Ct method (Bulletin #2: ABI Prism 7700 Sequence Detection System, Applied Biosystems, 1997).

## Results and discussion

Early detection is a major factor contributing to the 2.3% annual decline in breast cancer death rates over the past 10 years [17]. Nonetheless 40,480 women in the U.S. were projected to die from breast cancer in 2008 [18], in part because currently available breast cancer screening tools such as mammography and breast examination miss 10–40% of early breast cancers and are least effective in detecting cancer in young women, whose tumors are often more aggressive. An invasive needle or surgical biopsy must be performed when an area of suspicion is identified in order to confirm, by cytologic or histologic evaluation, the presence of malignancy, even though 66–85% of abnormalities are benign [19].

In principle, there are three different ways in which biomarkers can be used. First, they can be used to differentiate normal from diseased states. Second, they can be used to determine into which prognostic groups subjects should be placed. Finally, biomarkers can be used to monitor response to therapy. The identification of biologic markers such as miRNA species collected noninvasively in the circulation that would distinguish between women with or without breast cancer is therefore of crucial importance in early cancer detection, to improve disease free and overall survival from the disease. There is a recent report which outlines the usefulness of miRNA signatures to distinguish normal from diseased tissues and tissue origin, relying upon miRNA signatures from formalin fixed, paraffin embedded and frozen tissue specimens [20], demonstrating the stability of miRNAs. Our miRNA findings in archival serum support and extend these findings, with the potential that miRNA analysis may prove useful for noninvasive breast cancer detection.

**Figure 1:**
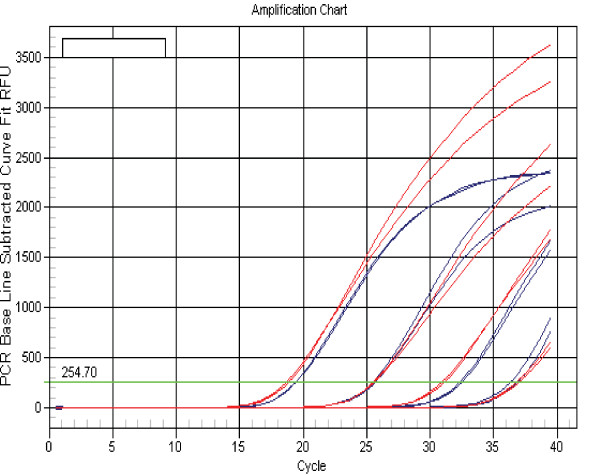
**Different quantities (10^5^, 10^3^, 10, 10^-1^, and 0 pg) of cel-miR-39 (blue curves) and has-miR-145 (red curves) inputs led to similar corresponding Ct values of 19.2, 25.8, 32.4, 37.10 and undetectable, respectively**.

**Figure 2:**
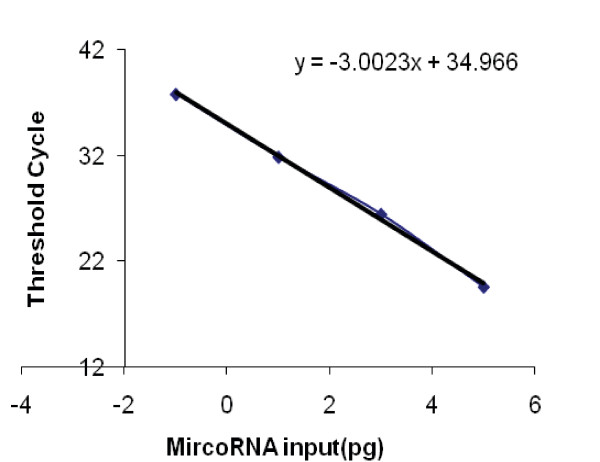
**There was linear recovery (slope = -3.0) of the miR-39 and miR-145 at concentrations equal to and greater than 10^-1^pg/400 μL serum**.

**Figure 3:**
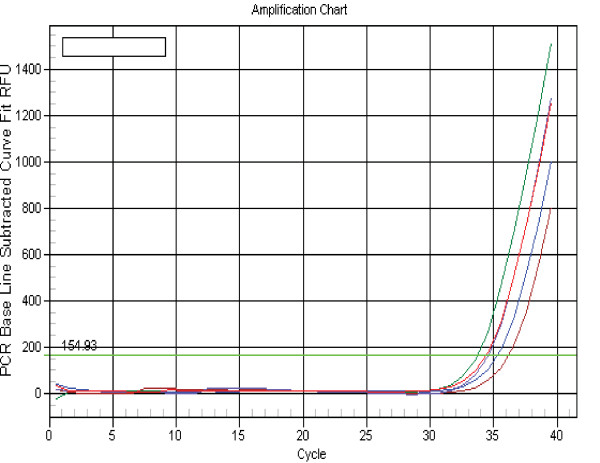
**The Ct value for endogenous GAPDH mRNA in each of the serial human serum samples to which miR-145 was spiked in was approximately 35**.

We first tested if spiked-in cel-miR-39 and has-miR-145 were detectable in a 10 year old human serum and a goat serum specimen. The specimen was divided into five samples of equal volume. Decreasing inputs of single-strand cel-miR-39 and has-miR-145 (10^5^, 10^3^, 10, 10^-1 ^and 0 pg) added to each sample (Figures 1 and 2) led to the corresponding mean Ct values (19.2, 25.8, 32.4, 37.1 and 38.7), respectively, with similar values for cel-39 and has-145. The Ct value of 37.1 obtained using the most dilute sample (10^-1 ^pg) was slightly lower than background of 38.7, indicating that the level of endogenous miR-145 in the human serum to which the synthetic single stranded miR-145 mimic was spiked in was very low, and therefore unlikely to have influenced the amplification of the synthesized miR-145. The equidistant curves down to a concentration of 10^-1^pg/400 μL serum suggest linear recovery (Figure 1) and efficient amplification (Figure 2, slope = -3.0) of the added miRNAs. The Ct values (Figure 3) for endogenous GAPDH mRNA in the serial serum samples to which has-miR-145 was spiked in were approximately 35. The results demonstrate that circulating RNA, including endogenous GAPDH mRNA and spiked-in cel-miR-39 and has-miR-145, can be efficiently extracted and amplified from serum.

We then performed a pilot study in serum specimens from 21 women with and without breast cancer (Table 2) to assess the expression of miR-16, -145 and -155 using 18s rRNA to normalize the relative expression of the miRNAs in archived serum specimens from women with and without breast cancer. Each miRNA and 18s rRNA was isolated and amplified efficiently in all subjects. The expression of all three miRNAs in serum was similar in samples from healthy women compared to those with breast cancer, unlike a prior report which found differences in the expression of mi-R-145 and -155 miRNAs in breast cancer tissue vs. benign tissue women [6]. On the other hand, progesterone receptor (PR, p = 0.016) positive tumors had higher miR-155 expression than tumors that were negative for this receptor. miR-155 has been classified as a multifunctional miRNA, having an important role in both normal and pathologic processes in immunity, inflammation, cancer and cardiovascular disease [21]. The significance of our finding regarding miR-155 and the PR will require a larger study.

**Table 2:** The Ct Value of miR-16, miR-145 and miR155 in the Serum Samples with and without Breast Cancer

***Ct value***	***miR-16***		***miR-145***		***miR-155***	
	NL^1^	TU^1^	NL	TU	NL	TU

Range	21.57–25.55	21.76–26.14	32.96–36.50	32.62–37.60	33.95–35.84	31.88–38.94

Average	23.75	24.23	34.90	35.51	34.87	34.98

Median	23.79	24.46	34.97	35.35	34.93	34.90

T-test	0.49		0.35		0.87	

1: NL = Normal serum, TU = Tumor serum. 21 serum samples (13 from subjects with breast cancer and eight controls). 18s rRNA was used to normalize the relative expression of miRNA species.

In determining the proper endogenous control for qPCR, we initially tried three small non-coding nucleoar RNAs (Rnu44, Rnu48 and Rnu66). Unfortunately, none were useful because we could not detect them. GAPDH was also tested but relatively high Ct values were observed, so 18s rRNA was tried levels adequate to serve as a control in all human serum samples. We confirmed that our techniques yielded adequate RNA extraction efficiency by measuring levels of spiked-in cel-miR-39 and has-miR-145. The spiked-in miRNAs had the advantage of lacking homology to and thereby interference from endogenous human miRNAs.

## Conclusion

The efficient extraction and accurate identification of miRNAs from serum represents a key first step toward the development of a noninvasive, blood-based detection test for breast cancer, and we believe represents an important advancement in achieving this goal.

## Abbreviations

ER: estrogen receptor; mi-, m- and rRNA: micro-, messenger and ribosomal ribonucleic acid; Rnu: nucleolar RNA; qPCR: quantitative polymerase chain reaction; PR: progesterone receptor; RT: reverse transcription.

## Competing interests

The authors declare that they have no competing interests.

## Authors' contributions

All authors read and approved the final manuscript. WZ and WQ helped conceive the project, designed and performed the experiments, UA helped design the project, provided scientific and technical insight and manuscript review, and ERS provided the samples, helped conceive the project, prepared the manuscript, provided scientific and clinical input, and submitted the manuscript.
